# Pure Red Cell Aplasia after 13 Years of Sodium Valproate, and Bone Marrow Suppression after 17 Years of Carbamazepine

**DOI:** 10.1371/journal.pmed.0010051

**Published:** 2004-11-30

**Authors:** Tiong The, Ratnavalli Kolla, Fitzroy Dawkins, Annapurni Jayam Trouth

**Affiliations:** **1**Case report fromSaint Peter's University Hospital, New BrunswickNew JerseyUnited States of America; **2**Howard University Hospital, WashingtonDistrict of ColumiaUnited States of America

## Abstract

A 38-year-old woman presented with acute hematological toxicity from her anticonvulsants, even though she had been taking them for many years

## PRESENTATION of CASE

**Figure 1:**
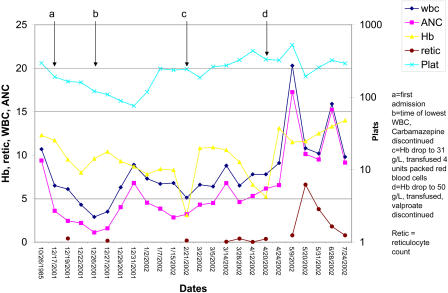
Patient's Clinical Course, Complete Blood Count, and Reticulocyte Count The x-axis shows specific dates. In 1985, prior to starting anticonvulsants, the only abnormality was an MCV of 106 fl. On admission (a), in 2001, the only abnormality was an increased MCV of 119 fl. During the second week of admission (b), her WBC, then her platelet count, dropped to their lowest levels, while her Hb showed a gradual decline. Bone marrow biopsy showed suppression of all marrow elements. After stopping carbamazepine, there was a brisk recovery of her WBC and platelet counts. Six weeks later (c), in February 2002, her Hb had dropped to 31 g/l, and she was given a transfusion of packed cells. Eight weeks later (d), in April 2002, despite erythropoietin and steroid therapy, her Hb dropped to 53 g/l and she received another transfusion. At this time, the sodium valproate was stopped. The reticulocyte count had remained abnormally low throughout this period (a–d), and it was only after stopping the valproate that the reticulocyte count and Hb started to rise. Her MCV dropped after the first transfusion and did not rise again until there was a brisk reticulocyte response. ANC, absolute neutrophil count; retic, reticulocytes; Plat(s), platelets.

A 38-y-old woman with Down syndrome was admitted to hospital for investigation of a 6-mo history of anorexia and weight loss of 40 lbs. Six months prior to admission, her weight was 175 lbs, and her body mass index was 36. On admission, her complete blood count was normal, but over a 2-wk period she developed acute pancytopenia (Figure 1). During this acute episode, her lowest hematological parameters were as follows: hemoglobin (Hb), 80 g/l (normal range 120–160 g/l); mean cell volume (MCV), 121 fl (80–95 fl); white blood cell count (WBC), 2.9 × 10^6^/l (4.8–10.8 × 10^6^/l); absolute neutrophil count, 1.1 × 10^6^/l (1.5–6.2 × 10^6^/l); and platelet count, 76 × 10^6^/l (150–350 × 10^6^/l).

She had been taking carbamazepine for 17 y and sodium valproate for 13 y for a mixed seizure disorder. At age 22 y, before starting any anticonvulsants, her baseline hematological parameters were as follows: Hb,123 g/l; MCV, 106 fl; platelets, 296 × 10^6^/l; and WBC, 10.7 × 10^6^/l. After starting carbamazepine, her WBC dropped to 4.5–5.5 × 10^6^/l, and her absolute neutrophil count dropped from 9 × 10^6^/l to about 2.5 × 10^6^/l. When the sodium valproate was added, her MCV increased to 112 fl, her platelet count fell to 100–150 × 10^6^/l, and her Hb dropped to 110–120 g/l.

The patient's other medications on admission were carnitine and low-dose L-thyroxine for hypothyroidism; she had been taking both for several years. She had not been recently exposed to any new medications, environmental toxins, or over-the-counter dietary supplements. There was no family history of aplastic anemia. The patient lived at home with her mother, who cares for her and has legal guardianship.

**Figure 2:**
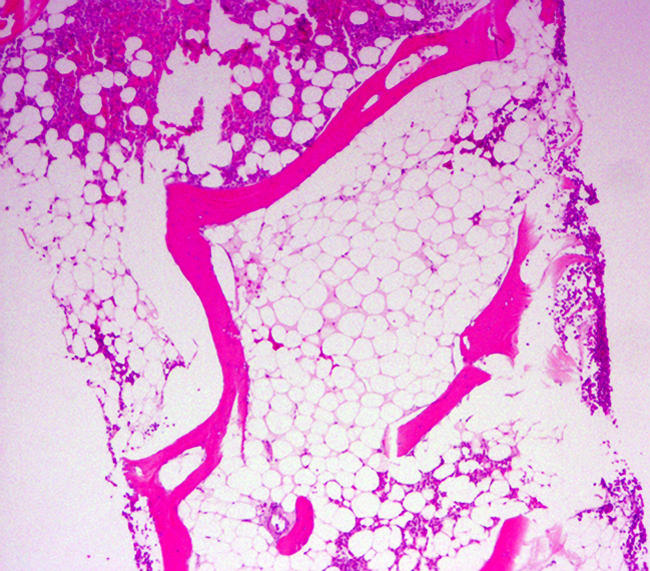
Bone Marrow Biopsy on Admission, Showing Predominantly Hypocellular Bone Marrow

A bone marrow biopsy (Figure 2) showed a hypocellular bone marrow with a normal myeloid:erythroid ratio and no malignant cells or megaloblastic changes. Cytogenetic study showed a Robertsonian (i.e., of the whole arms) translocation of Chromosome 14 and 21 involving the long arm of Chromosome 21. Except for mild adrenal insufficiency noted on an adrenocorticotropic hormone stimulation test, all her tests, including viral and immunological investigations to identify known causes of aplastic anemia, were negative. The patient's thyroid function tests were normal. She was started on low-dose hydrocortisone for her adrenal insufficiency and megestrol acetate to increase her appetite.

The patient's carbamazepine was discontinued. Within 10 d, her WBC had risen to 6.7 × 10^6^/l and her platelet count to 248 × 10^6^/l. Serial complete blood counts showed worsening anemia over the next 15 wk, requiring two packed red blood cell transfusions at week 6 and week 14, when her Hb was 31 and 53 g/l, respectively. Throughout this period, her reticulocyte count was low, at about 0.18% (normal range 0.5%–2%), while her WBC and platelet count were normal. We made a diagnosis of pure red cell aplasia (PRCA). A second bone marrow biopsy under minimally conscious sedation was unsuccessful, and the patient declined further attempts. After the first transfusion she was started on prednisone and erythropoietin for her PRCA. Meanwhile, because of her worsening seizures, we increased her sodium valproate dose, increasing her serum valproate level from 70 mcg/ml to 110 mcg/ml. After the second transfusion, we discontinued her sodium valproate and started her on clonazepam and oxcarbazepine as alternative anticonvulsants.

The patient had a brisk reticulocyte response. Her reticulocyte count rose from 0.36% to 0.78% in the second week and to 6.61% in the fourth week after stopping the valproate. Six weeks after stopping the drug, her Hb was 125 g/l and her MCV was 106 fl, suggesting replacement of transfused red blood cells with newly formed red blood cells (which have a higher MCV than older transfused cells). Over the next 30 mo of follow-up, she had no relapse of her aplastic anemia.

We were unable to identify a specific cause for the patient's anorexia and weight loss. We found no evidence of malignancy on admission or subsequent follow-up. Within 1 wk of stopping her sodium valproate, her appetite improved and she put on 15 lbs over the next 6 wk. By the fourth month after stopping the drug, she had gained 45 lbs.

We last saw the patient in August 2004. Her seizure control had worsened, and she had developed signs of early dementia. Her current anticonvulsants are clonazepam, oxcarbazepine, and zonisamide, and she continues taking synthroid. Her last complete blood count was stable: WBC, 6.4 × 10^6^/l; Hb, 147 g/l; MCV, 104.9 fl; and platelets, 262 × 10^6^/l.

## Discussion

Sodium valproate and carbamazepine are associated with rare but potentially lethal hematological complications. There are five case reports of PRCA shortly after initiation of sodium valproate, with the longest interval between the initiation of therapy and the onset of aplasia being 2 y [1,2,3,4,5]. Acute bone marrow suppression with leucopenia and thrombocytopenia associated with carbamazepine most often occur within 4 mo of starting treatment [6,7]. As far as we know, our case report is unique in that the patient developed acute bone marrow suppression and PRCA after 17 y of carbamazepine and 13 y of sodium valproate therapy.

Our investigations ruled out most of the known causes of acute bone marrow suppression, making the anticonvulsants the most likely cause. Malnutrition was an unlikely cause: her WBC and platelet counts had recovered before any increase in appetite or weight gain; her body mass index was normal despite her weight loss; and her bone marrow iron stores were adequate and her serum folate and vitamin B12 levels were high, suggesting adequate nutrient supply.

We considered and rejected the possibility of Down syndrome–associated aplastic anemia. There have been six case reports of this condition, and in all cases it occurred in young children, suggesting a genetic predisposition [8]. Half of these patients died, and half responded partially to androgenic steroids. Our patient was an adult, and her bone marrow had responded to the withdrawal of her anticonvulsants and not to the administration of androgenic steroid. She had failed to respond to a 6-wk course of high-dose non-androgenic steroid and erythropoietin. Furthermore, our patient did not have a relapse of her aplastic anemia in 30 mo of follow-up, suggesting a lack of genetic disposition.

The brisk return of her WBC and platelet counts upon discontinuing carbamazepine, and her brisk reticulocytosis upon discontinuing sodium valproate, were both consistent with previous reports of hematological toxicity due to these drugs [1,2,3,4,5,6,7]. What was unusual in our case was the extremely late onset of bone marrow suppression after initiation of drug therapy. The persistent suppression of erythropoietic elements for 15 wk after stopping the carbamazepine was unlikely to be due to persistent residual marrow suppression by carbamazepine, since carbamazepine-induced PRCA responds quickly (within 1–2 wk) to stopping the drug [6,9]. We believe that the continued use of sodium valproate, after stopping the carbamazepine, caused the persistent suppression of erythropoietic elements.

We found no cause for the patient's anorexia and weight loss, but her appetite returned and she gained weight after stopping the sodium valproate. There is a known association between this drug and anorexia [10].

**Learning Points**

- Acute hematological complications of anticonvulsant therapy can still occur after many years of therapy, so continued vigilance is warranted.
- Although sodium valproate is generally associated with increased appetite and weight gain, it can also be associated with anorexia, nausea, vomiting, and weight loss.

## Abbreviations

Hb: hemoglobin
MCV: mean cell volume
PRCA: pure red cell aplasia
WBC: white blood cell count
